# Quantification of ductal blood flow with magnetic resonance imaging in newborns with obstructive left heart disease

**DOI:** 10.1186/1532-429X-14-S1-P118

**Published:** 2012-02-01

**Authors:** Marcelo F Kozak, Luc Mertens, Ashley Ho, Shi-Joon Yoo, Lars Grosse-Wortmann

**Affiliations:** 1 Section of Cardiac Imaging, Division of Cardiology, The Hospital for Sick Children, University of Toronto, Toronto, ON, Canada

## Background

Patients with congenital obstructive left heart lesions often depend on ductal blood flow to supplement their systemic circulation. Echocardiography (ECHO) is routinely used to assess duct patency and shunt direction, but may be of limited value in shunt quantification. We hypothesized that ECHO assessment of net direction and magnitude of blood flow across a patent ductus arteriosus (PDA) is unreliable as compared to cardiac magnetic resonance (CMR).

## Methods

From 2003 to 2011, 40 newborns with obstructive left heart lesions had undergone ECHO and MRI prior to any intervention to alleviate the obstruction. Twelve out of these 40 patients matched the inclusion criteria: 1) on prostaglandin infusion, 2) ECHO and MRI performed within 2 days, and 3) oxygen saturation difference between the two tests within 10%. We retrospectively compared the shunt quantification across the PDA by both methods: Phase contrast flow velocity mapping by CMR and velocity time integral (VTI) by ECHO. Age ranged between 0 and 9 days; other characteristics are detailed in the table.

**Table 1:** Demographics and measurements by MRI and echocardiography

Patient	Weight (Kg)	Height (cm)	BSA (m2)	Diagnosis	PDA diameter (cm) (MRI)	PDA diameter (cm)(ECHO)	Flow direction(MRI)	Flow direction (ECHO)	Net flow (L/min/m2) (MRI)	Net flow (L/min/m2) (ECHO)
1	3.3	50	0.21	HLHS	0.38	0.4	1	0	1.7	0.66
2	2.5	44	0.17	Critical AoV stenosis	0.56	0.31	1	1	0.56	0.26
3	3.3	49	0.21	HLHS	0.66	0.6	1	1	2.53	0.63
4	2	48	0.16	Shone's	0.66	0.3	1	1	2.18	0.62
5	3.5	50	0.22	CoA	0.85	0.72	1	1	1.64	0.96
6	3	47	0.2	Tumor in LV	0.71	0.68	0	1	0.81	0.09
7	2.8	40	0.18	HLHS	0.48	0.49	1	1	1.22	1.7
8	3.3	51	0.22	Shone's	0.66	0.55	0	1	0.82	1.69
9	1.7	40	0.14	HLHS	0.76	0.57	1	1	1.1	0.16
10	3.3	50	0.21	Shone's	0.77	0.48	1	1	0.05	0.36
11	3.7	46	0.22	Shone's	0.66	0.62	1	1	0.67	1.04
12	4	53	0.24	Shone's	0.75	0.74	1	0	1.19	0.6

PDA: patent ductus arteriosus; HLHS: hypoplastic left heart syndrome; AoV: aortic valve; flow direction: 0 = left-to-right; 1 = right-to-left.

## Results

There was no significant difference between net flow measurements by CMR or ECHO-VTI (p = 0.18). However, there was poor agreement among methods with a wide confidence interval (figure) and a low intra-class correlation coefficient of -0.29. Also, when comparing CMR measurements with a semi-quantitative analysis by an experienced echocardiographer, we found that the net flow direction was estimated correctly by echocardiography in 2/3 of cases. The correlation between visual flow quantification by echocardiography (mild, moderate, severe) and CMR net flow in the 8 patients rated to have a net right-to-left shunt by both methods was poor (Kendall’s rank correlation coefficient 0.13, p NS). PDA net right-to-left shunt by CMR correlated with the ratio of systolic to diastolic flow duration (Rho = 0.66; p = 0.02), but not with oxygen saturation (Rho = -0.45; p = 0.14); age (Rho = 0.27; p = 0.39), PDA diameter (Rho = -0.22; p = 0.5), and heart rate (Rho = -0.08; p = 0.8).

**Figure 1:**
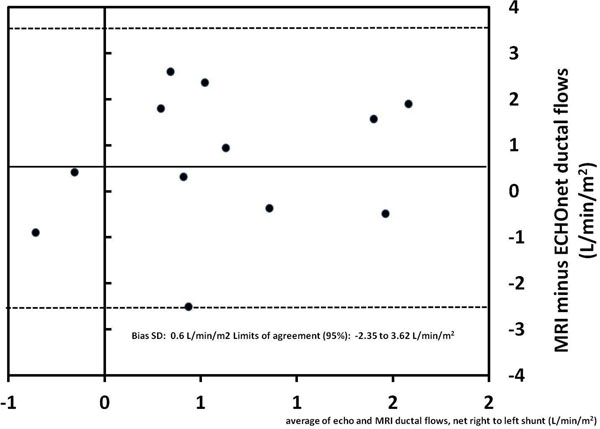


## Conclusions

In patients with obstructive left heart lesions, ECHO based estimates of shunt magnitude across a PDA are unreliable. This is true for semiquantitative visual assessment and for flow estimation by VTI. The presence of a large caliber PDA does not imply the presence of a large amount of net shunting. A longer relative duration of systole allows for more ductal right-to-left shunting.

## Funding

None.

